# Efficacy and Accuracy of Ultrasound Guided Injections in the Treatment of Cervical Facet Joint Syndrome: A Systematic Review

**DOI:** 10.3390/jcm13175290

**Published:** 2024-09-06

**Authors:** Mattia Giuseppe Viva, Valerio Sveva, Marco Ruggiero, Annatonia Fai, Alessio Savina, Riccardo Perrone, Danilo Donati, Roberto Tedeschi, Marco Monticone, Giacomo Farì, Andrea Bernetti

**Affiliations:** 1 Department of Anatomical and Histological Sciences, Legal Medicine and Orthopedics, Sapienza University, 00189 Rome, Italy; mattiagiuseppe.viva@uniroma1.it (M.G.V.); valerio.sveva@uniroma1.it (V.S.); alessio.savina@uniroma1.it (A.S.); 2 Physical and Rehabilitation Medicine 2, IRCCS Istituto Ortopedico Rizzoli, 40136 Bologna, Italy; marco.ruggiero@ior.it; 3 Department of Translational Biomedicine and Neuroscience (DiBraiN), Aldo Moro University, 70121 Bari, Italy; a.fai1@studenti.uniba.it; 4 Unit of Physical Medicine and Rehabilitation, Sant’Andrea Hospital, “Sapienza” University of Rome, 00189 Rome, Italy; perrone.1391224@studenti.uniroma1.it; 5 Clinical and Experimental Medicine PhD Program, University of Modena and Reggio Emilia, 41125 Modena, Italy; 6 Department of Biomedical and Neuromotor Sciences, Alma Mater Studiorum University, 40126 Bologna, Italy; roberto.tedeschi2@unibo.it; 7 Department of Surgical Sciences, University of Cagliari, 09124 Cagliari, Italy; marco.monticone@unica.it; 8 Department of Experimental Medicine (Di.Me.S.), University of Salento, 73100 Lecce, Italy; andrea.bernetti@unisalento.it

**Keywords:** chronic neck pain, cervical facet joint, facet joint injection, ultrasound-guided injection, medial branch block, third occipital nerve block

## Abstract

**Background/Objectives**: Cervical facet joint syndrome (CFJS) is a frequent cause of neck pain and motor disability. Among the available therapies for CFJS, ultrasound (US)-guided injections are becoming more and more widespread, but the evidence about their accuracy and effectiveness is still debated in the scientific literature. The aim of this systematic review is to assess efficacy, accuracy and feasibility of US-guided cervical facet injections for the related chronic neck pain treatment. **Methods**: This review was conducted following the preferred reporting items for systematic reviews and meta-analysis 2020 (PRISMA) statement guidelines. The scientific articles were identified through the PubMed, Google Scholar and Cochrane Library databases. Qualitative assessment of the selected studies was carried out using the modified Oxford quality scoring system. Nine studies with a total of 958 patients were included in this review. The risk of bias was assessed using the Cochrane Collaboration tool. The protocol was registered at PROSPERO 2024 (n°CRD42024512214). **Results:** The results of this review suggest that the US-guided cervical facet injection for CFJS treatment is an effective technique in terms of accuracy (using the lateral technique it ranges from 92% to 98%), and efficiency (it grants pain relief with a decrease in the procedure time and fewer needle passes in comparison with the X-ray-guided technique, which also involves radiation exposure). **Conclusions:** US-guided injections are a safe and effective method to treat this musculoskeletal disease, granting a high functional recovery and long-lasting pain relief, net of the used drugs. However, these procedures are strictly operator-dependent and require important training to acquire good expertise.

## 1. Introduction

Cervical pain is one of the most common conditions requiring healthcare treatment in industrialized countries [1]. Each year, neck pain affects 30–50% of the general population [2]. It is estimated that 15% of the population will experience chronic neck pain (lasting longer than three months) at some point in their lives, with its prevalence increasing with age [3]. According to the 2024 report by the Global Burden of Diseases, Injuries, and Risk Factors Study (GBD), neck pain affected 203 million people between 1990 and 2020 [4]. By 2050, the global number of neck pain cases is projected to rise to 269 million, representing a 32.5% increase from 2020, particularly in low- and middle-income countries, driven by a rapidly aging global population [4]. Moreover, females have a higher age-standardized prevalence rate (2890 per 100,000) compared to males (2000 per 100,000), with the prevalence peaking between the ages of 45 and 74 in both sexes [4]. Interestingly, the age-standardized disability-adjusted life years (DALYs) rate for neck pain decreased globally from 1990 to 2019, largely due to improved management and the development of new treatments [5]. As a result, chronic neck pain can lead to severe motor disability, which is expected to have significant social and economic consequences worldwide in the coming decades, continuing to pose a major challenge in terms of disability burden globally [4].

Among the main causes of chronic neck pain, cervical facet joint syndrome (CFJS) plays a fundamental role [6]. It is characterized by the presence of neck pain, often radiating to the head, back and upper extremities, particularly the shoulders, and causing a drastic limitation in the neck’s range of active motion (ROM) [7]. This syndrome occurs when the facet joints undergo biomechanical dysfunction and cartilaginous degeneration, especially as a consequence of osteoarthritis, hypertrophied superior articular process and facet joint cysts [8]. In fact, cervical facet joints are delicate diarthrodial joints formed by the articulation between the superior articular process of one vertebra and the inferior articular process of the vertebra above, located at the junction of the lamina and pedicle [9]. These facet joints, along with their capsule, were shown to contain nociceptive elements, which are believed to act as independent pain generators and may contribute to chronic neck pain in an estimated 35% to 55% of cases [10].

Various treatments were proposed in pain medicine and rehabilitation for the management of CFJS, including radiofrequency ablation [11], physiotherapy [12], therapeutic exercise [13], and cervical manipulations [14]. In recent years, interventional procedures have emerged as a particularly effective therapy. The American Society of Interventional Pain Physicians (ASIPP) included both radiofrequency and facet joint steroid or anesthetic injections as recommended treatments for CFJS in their 2020 Cervical Interventions Guidelines [15]. However, the success of radiofrequency was linked to stringent selection criteria, which are associated with high success rates but also an increased rate of false-negative results due to strict diagnostic requirements.

A more recent consensus practice guideline from a multispecialty international working group has highlighted some limitations of using radiofrequency ablation as a first-line treatment for CFJS [16]. While radiofrequency ablation is a complex technique requiring expensive medical equipment, specific settings like operating rooms dedicated to interventional procedures, and highly trained physicians, diagnostic and therapeutic facet joint nerve blocks, as well as intra-articular injections, are simpler and less expensive options. These procedures can be performed under ultrasound guidance, which minimizes the risks associated with procedural interventions, such as bleeding complications and injury to aberrant vasculature and surrounding nerves (e.g., cervical nerve roots), which are more prevalent in the cervical spine compared to the thoracic or lumbar spine [16].

As mentioned above, the injection techniques that can be carried out under US guidance are the following: the cervical facet injections, the cervical medial branch or the third occipital nerve (TON) blocks [17]. With regards to the cervical facet injections, the patient is positioned in the lateral decubitus position, with his head resting comfortably on a pillow and the side of interest facing upward. The US transducer is placed over the lateral side of the neck in the axial plane to start visualizing the posterior tubercle of the segmental foramina [17]. Once the target facet joint is identified, the needle is advanced in-plane using an anterior-to-posterior approach and the drugs are injected in the intra-articular space. Concerning the cervical medial branch blocks and the TON blocks, similarly to radiofrequency, these procedures are directed to the medial branch of the nerve responsible for the pain sensitivity of the facet that causes the syndrome to be treated [17]. In these cases, the patient is positioned on his side in the lateral decubitus position, with the head comfortably supported by a pillow and the side of interest facing upward. The US transducer is placed in a coronal plane with the cephalic end on the mastoid process. Then, the transducer must be moved until it can be visualized as the articular pillar of C2. Next, translating caudally until the C2-C3 articulation, the TON can be visualized [17]. In order to visualize the caudal medial branches, the transducer must be moved caudally in a coronal view. In this way, articular processes and joints are visualized as hyperechoic peaks with a cleft, while the hyperechoic valleys indicate the location of the medial branches [17]. Using an out-of-plane approach, a short 25-gauge needle can be advanced from anterior to posterior, aiming for the deepest point along the near-contiguous hyperechoic bony ridge. Then, steroids and anesthetics can be easily injected.

As said before, both of these techniques are gaining more and more importance due to their ease and effectiveness in clinical practice, especially because they do not require exposure to ionizing radiation. Nevertheless, the level of scientific evidence of the cervical facet injections in terms of efficacy and safety is still debated in the available literature, as there is a lack of sufficient and solid data regarding their accuracy, effectiveness and feasibility, with particular regard to the new US-guided procedures, for which the evidence, although very promising, still seems confused and fragmentary. Therefore, the aim of this systematic review is to assess the efficacy, accuracy and feasibility of the US-guided cervical facet injections for CFJS treatment, bringing order to the existing evidence, highlighting their limitations and advantages, and thus offering useful recommendations and insights to the health professionals involved in the clinical management of CFJS.

## 2. Materials and Methods

### 2.1. Data Sources, Search Strategy and Study Selection

This review adhered to the Preferred Reporting Items for Systematic Reviews and Meta-Analysis (PRISMA) statement guidelines [18]. The protocol was registered at PROSPERO 2024 (n°CRD42024512214). The scientific articles were identified through PubMed, Google Scholar, Scopus, Web of Science and Cochrane Library databases, using the Medical Subject Headings (MeSH) as applicable. The eligible studies were identified by using the following Boolean search syntax: “(“Cervical” AND (“facet joints” OR “Zygapophyseal joints” OR “Medial branch”) AND (“injection” OR “block”) AND (“ultrasound”/“CT” /“fluoroscopy”/“pain management”)”. Afterward, the following filters were activated—text availability: full text; species: humans; languages: English; period: last ten years (2013–2023). In order to clarify the time frame selection, we chose to analyze the last ten years because the majority of the literature on this topic was published during this period, and because RCTs on ultrasound-guided interventions were not published until after 2013. The search syntax used for the PubMed database was a mix of MeSH database and Boolean search syntax. The references of the articles were manually examined to find the more relevant publications. Once potential articles were selected, they were further filtered based on specific criteria for inclusion or exclusion. Inclusion criteria were as follows: (1) patients aged >18 years who received US- or Fluoroscopic (FL)-guided intra-articular injection or Cervical Medial Branch Blocks (CMBBs) for the treatment of CFJS; (2) pain lasting for at least 3 months; (3) patients not responder to conservative management, including analgesic or physical therapy; (4) studies with outcome measures including pain intensity changes measured by VAS or NRS scales, Patient-Reported Outcome Measures (PROMs) questionnaires, cervical active and passive range of motion (ROM); (5) studies including measures of interventional accuracy rate, safety and percentage of side effects during and after the procedure with clinical follow-up; (6) studies published between 1 January 2013 and 31 January 2024. Exclusion criteria concerned studies such as comments, expert opinions, case reports, case series, conference meeting abstracts, surveys, reviews, editorials, systematic reviews, meta-analyses, and letters.

### 2.2. Data Extraction and Outcome Measures

Six investigators (M.V., M.R., V.S., A.S, A.F., R.P.) independently assessed each title, abstract, and full-text article for eligible studies. Disagreements were resolved by consensus by asking other four separate experienced investigators (A.B., G.F., M.M., D.D.). By means of data extraction forms, the information was obtained from each study by including the type of intervention, the injected drugs, the population characteristics (number of participants, age and sex), the use of US or FL guidance, the analyzed outcomes and their respective follow-up periods. The outcome measures of interest were: (1) pain measures as the Visual Analogue Scale (VAS) or the Numerical Rating Scale (NRS); (2) clinical symptoms and functional features as motor measures (e.g., cranio-cervical flexion test, pressure pain thresholds); (3) PROMs questionnaires and subjective assessment of treatment results; (4) valuation of the accuracy of the intervention through cervical spine computed tomography (CT), to evaluate the presence of the injected substance at the target site after the procedure. Main statistics and effect estimates were reported for each outcome.

### 2.3. Quality Assessment

The selected studies underwent a qualitative assessment using the Modified Oxford Quality Scoring System, which is also referred to as the Modified Jadad Score [19]. The Modified Jadad Score is a four-question scale that evaluates randomization and concealment of treatment allocation groups, withdrawals and dropout rates, adherence to inclusion and exclusion criteria, and clarity in describing statistical methods within the studies under analysis. Each aspect was assessed independently by the six investigators mentioned earlier. The Modified Jadad score ranges from 0 to 5 and each question has a dichotomic answer (yes—1 point; no—0 point) [20]. Higher score indicates a better study quality. If a study had a modified Jadad score >3 points, it was of high quality; if the score was 2–3 points, it was of moderate quality; and if the score was <2 points, it was of low quality. As stated by Olivo et al. [19], the Modified Jadad Score demonstrated the best evidence for validity and reliability in quality assessment in this field. However, there is still a need to develop a valid and reliable scale specifically for assessing the methodological quality of trials.

### 2.4. Risk of Bias Assessment

The risk of bias for all the included studies was assessed evaluating the six domains defined by the Cochrane Collaboration tool [21]. These six domains are (1) selection bias due to random sequence generation and allocation concealment; (2) performance bias, with blinding of participants and personnel as a possible source of bias; (3) detection bias due to blinding of the outcome assessment; (4) attrition bias, evaluating possible incomplete outcome data; (5) reporting bias due to selective outcome reporting; and (6) other bias, evaluating any important concerns about bias not covered in the other domains. Each domain was judged as “low risk of bias” (“green”), “high risk of bias” (“red”), or “unclear risk of bias” (“yellow”). This systematic review adheres to the Cochrane Collaboration criteria for evaluating all the studies retrieved [21]. All reviewers received training on the use of the Cochrane Collaboration tool, and one reviewer (M.M.), who has experience in developing Cochrane systematic reviews [22], contributed to the writing and analysis of this review.

Finally, visualization of the authors’ judgments about each risk of bias item was presented both as percentage and as summary across all the included studies using the Risk of Bias visualization tool [23].

## 3. Results

### 3.1. Identification of Studies

**Figure 1:**
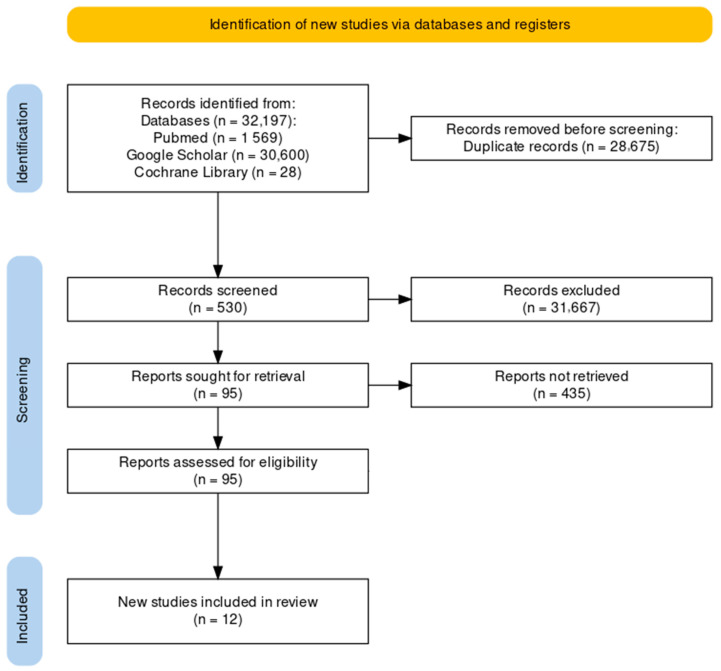
Study selection and eligibility screening flow according to PRISMA guidelines.

The studies were identified through a search of five databases (Pubmed, Google Scholar, Scopus, Web of Science and Cochrane Library). At the end of the selection process, 32.197 articles were extracted, of which 15.688 were from Pubmed, 5.832 were from Google Scholar, 3.456 were from Scopus, 3.387 were from Web of Science, and 3.834 were from the Cochrane Library. These articles were identified by searching for studies of likely relevance to the review. Duplicates were eliminated (*n* = 28.675). Thus, all the titles and abstracts were selected, removing review articles (*n* = 37), meta-analyses (*n* = 3), case reports (*n* = 2), letters (*n* = 7), editorials (*n* = 8), systematic reviews and meta-analysis (*n* = 8), cadaveric studies (*n* = 3), conference meetings (*n* = 2), and studies without accuracy and safety evaluations (*n* = 30). Subsequently, the full text of the remaining 95 articles was assessed to verify their eligibility. Finally, nine research articles were included (Figure 1) [13,24,25,26,27,28,29,30,31].

### 3.2. Characteristics of the Included Studies

Table 1 shows the characteristics of the included studies retrieved from the systematic review. Sample size, a brief resume about the research design, the collected data and outcomes, as well as results were analyzed in all of the nine studies.

Nine studies including a total of 958 patients were analyzed in this review. The selected studies focused on the treatment of cervical chronic pain due to CFJS through injective treatment such as the injection of the zygapophyseal joint, the anesthetic block of the medial branch of the corresponding spinal nerve that innervates the facet joints themselves, and the TON block. All of the retrieved studies evaluated the effectiveness of the drugs used. Other studies compared the efficacy of different injection techniques and the use of fluoroscopic, TC or ultrasound guidance.

### 3.3. Anaesthetics and/or Corticosteroid Used in the Trials

Anaesthetics were used in all of the analyzed studies. Among these, bupivacaine, lidocaine and ropivacaine were shown to be equally effective in improving symptoms both when injected inside the joint and at the perineural level [13,24,25,26,27,28,29,30,31]. In four of these studies, the anesthetic is also associated with a corticosteroid [triamcinolone acetonide 40 mg/mL, dexamethasone (5 mg/mL) or betamethasone (4 mg/mL)] with an equally therapeutic success [13,24,28,29]. Pasuhirunnikorn et al. also compared the use of lidocaine vs. bupivacaine in cervical median branch block (CMBB) in 62 patients [30]. Although both were shown to be effective in improving symptoms, patients treated with lidocaine had a longer duration of analgesic effect and functional benefit. The volume of anesthetic varies depending on the study from 0.5 to 2.5 mL. For instance, Cohen et al. tried to reduce the volume of injected drug to 0.25 mL by evaluating its diffusion through CT of the cervical spine and were able to demonstrate how, with the same therapeutic effect, it reduces the aberrant diffusion of the drug, improving accuracy [25].

### 3.4. Efficacy, Accuracy, Performance Time and Pain Relief of US-Guided Cervical Facet Injections

In a randomized, observer-blinded trial, Finlayson et al. compared the TONB guided by US versus FL in terms of effectiveness and efficiency, using a 1:1 mixture of Omnipaque 240 (an iodinated contrast agent) and bupivacaine 0.75%. The study found that while both methods achieved similar success rates in the treatment of neck pain, US-guided significantly reduced the performance time and required fewer needle passes, improving the efficiency of the procedure, with no adverse events [26]. The same research group in 2015 carried out another randomized trial comparing US versus FL guidance for C7 CMBBs, focusing on performance time, success rate, pain relief, and procedural complications, injecting a 0.6 mL mixture of Omnipaque 240 and bupivacaine 0.75%. US guidance showed significantly shorter performance time and fewer needle passes while achieving the same success rates and pain relief obtained with FS [27]. In a retrospective comparative study carried out in 2016, Park et al. compared US- versus FL-guided CMBB in the management of chronic CFJS by infiltrating a mixture of 1% lidocaine (0.5 mL) and dexamethasone (5 mg/mL; 0.5 mL) [29]. Both the techniques had similar mid-term results in terms of pain relief, functional improvement and procedural efficiency over a 6-month period. The results suggest that although both US- and FL-guided offer significant pain relief and functional improvement, US-guided CMBBs are associated with shorter administration times and fewer needle passes. In a prospective randomized clinical trial conducted in 2013, Obernauer et al. randomized 40 adult patients into two groups to receive US- or CT-guided treatment. One mL of a mixture of betamethasone 4 mg/mL and bupivacaine hydrochloride 0.25%, 2.5 mg/mL was injected in all the patients [28]. Both groups showed significant benefit from the inter-articular injections 30 min and one-month post-injection: 30 min post-intervention there was an average reduction in VAS of 72% (one level) and 69% (two levels) in the US group compared to 50% (one level) and 52% (two levels) in the CT group. One month after the intervention, the US-guided showed an average reduction in VAS of 91% (one level) and 50% (two levels) compared with 59% (one level) and 55% (two levels) in the CT-guided. The study showed that US-guided injections may be preferable to minimize time and radiation exposure while preserving therapeutic efficacy [28].

### 3.5. Assessment of Methodology and Quality of the Studies

The methodological quality of the included studies was assessed using the Modified Jadad Score, as shown in Table 2. In this systematic review, two studies were identified as having high quality (scores > 4) [25,30], six studies were categorized as having moderate quality (scores 3 to 4) [13,26,27,28,29,31], while one study was identified as having low quality (scores < 3) [24].

### 3.6. Evaluation of Risk of Bias

**Figure 2:**
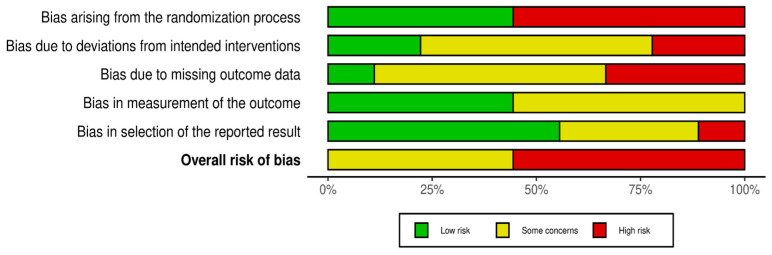
Risk of bias graph: review of the authors’ judgments about each risk of bias item presented as percentages across all the included studies.

The risk of bias graph is reported in Figure 2. The overall level of risk of bias in all of the studies selected in this systematic review showed some concerns about the randomization process (selection bias), deviations from the intended intervention (performance bias), blinding of the outcome assessment (detection bias), incomplete measurement of the outcome data (attrition bias), and selective reporting of the results (reporting bias). More specifically, 45.5% of the studies highlighted some concerns, while 55.5% of them had a high risk of bias.

**Figure 3:**
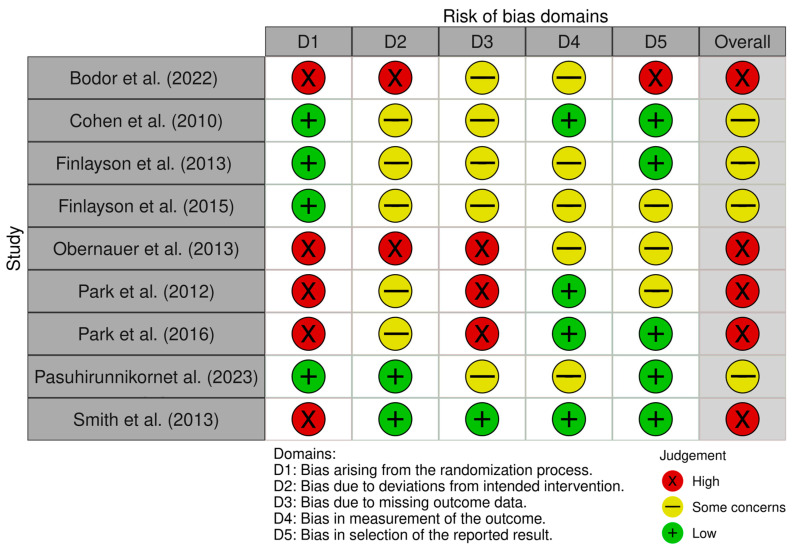
Risk of bias summary: review of the authors’ judgements about each risk of bias item for each included study [13,24,25,26,27,28,29,30,31].

A risk of bias summary is reported in Figure 3. It reveals that four out of the nine studies presented a low risk of bias [25,26,27,30] and five of them presented a high risk of bias arising from the randomization process [13,24,28,29,31]. Conversely, two of the nine studies showed a low risk of bias due to deviations from the intended intervention [30,31], and there were two studies with high risk [24,28]. Moreover, only one study had a low risk of bias in terms of missing outcome data [31], and three studies had a high risk [13,28,29]. Four studies revealed a low risk of bias in the outcomes measurement [13,25,29,31]. Lastly, five studies presented a low risk of bias in the selection of the reported results [25,26,29,30,31], and one had a high risk in this domain [24].

## 4. Discussion

This comprehensive systematic review aims to evaluate the efficacy and accuracy of ultrasound-guided (US-guided) cervical facet joint (CFJ) injections in patients suffering from chronic neck pain due to cervical facet joint syndrome (CFJS). The review examines both intra-articular techniques and medial branch block approaches, with the goal of providing useful recommendations for clinicians in their daily practice. A significant contribution to the field of interventional therapy for CFJS management is the emerging approach of using US-guided injections in carefully selected patients, focusing on its accuracy, safety, and reproducibility.

Currently, there is a substantial gap in the treatment of neck pain caused by CFJS, as highlighted by state-of-the-art guidelines and consensus papers. This systematic review also aims to address this gap in the literature by exploring and analyzing the various factors that need to be considered before, during, and after a CFJ injection.

In fact, these procedures are gaining more and more importance because they are a great option for CFJS treatments, and also due to the fact that they are often used as “diagnostic blocks”, which are useful for pain specialists in order to better identify the neck pain generator when there is a doubt if the underlying cause is linked to facet joints or to intervertebral discs. Therefore, a greater knowledge of the potentials and limits of these techniques according to the most recent scientific evidence is mandatory, similar to what has happened for other musculoskeletal diseases [32,33].

Facet joint infiltration is considered to be a low- to medium-risk procedure, with major complications rare and typically related to infection in predisposed patients [34]. Like any invasive procedure, it is necessary to discuss this information with the patient and have them sign an informed consent form. Complications can include bleeding, hematoma, septic arthritis, vasovagal reactions, vertebral artery damage and phrenic nerve palsy [35].

Complications such as swelling and pain at the needle insertion site usually resolve on their own and are short-lived. Severe reactions to local anesthetics are rare, and local reactions to steroid injections usually resolve within 48 h [36].

Even if it was not among the aims of this study, from the reviewed articles a good safety profile also emerges for all the US-guided procedures used for the treatment of CFJS. No significant side effects or reactions to US-guided procedures were reported. Relative contraindications to interventional techniques were described in patients receiving treatment with antithrombotic agents and anticoagulants. Prior to cervical facet joint interventions, patients on warfarin therapy must have their prothrombin time (PT) checked and documented to be at acceptable levels. The combination of multiple drugs with aspirin and nonsteroidal anti-inflammatory drugs or other antiplatelet therapy may be considered to increase the risk of spinal hematoma. Complications from intra-articular injections or medial branch blocks in the cervical spine (dural puncture, spinal cord trauma, subdural injection, neural trauma, epidural abscess and bacterial meningitis) are exceedingly rare in the related literature [37].

From this review, it clearly emerges that, when compared with FL- and CT-guided procedures, the advantages of the US-guided technique are notable. First of all, with equal effectiveness in terms of reducing neck pain and functional recovery, US guidance does not expose patients to ionizing radiation. In fact, all the considered outcome measures describe a high efficacy of the US-guided injections in the short and medium term. Net of the drugs used, the improvement obtained in the VAS and NRS scales, as well as the increase in cervical ROM and motor functions, are evident with US-guided techniques in all the selected studies. Moreover, in a retrospective study specifically aimed at comparing US-guided versus CT or FL-guided techniques involving 126 patients, US-guided injections with corticosteroid produced the same reductions in pain and disability scores lasting at least 6 months when compared with ionizing radiation-guide [38]. Therefore, US guidance grants also medium- to long-term effects, avoiding the risks of exposure to ionizing radiation for both patients and operators [39,40].

Then, this review highlights that all the US-guided procedures have the same accuracy as FL- and CT-guided ones, net of the injected drugs. Nevertheless, only US guidance allows real-time visualization of soft tissues too, so it is more reliable in reaching the anatomical target precisely and safely, without running the risk of damaging noble structures such as blood vessels. To further confirm this, two trials carried out on cadavers evaluated the accuracy of US guidance during injection. Galiano et al., in 40 injections on four cadavers, reported a high level of US accuracy in identifying and achieving the targeted joint space [41]. Similarly, Freire et al. obtained a 78% (31 of 40) US-guided facet joint injection success rate in 40 facet injections on four cadavers [42]. The accuracy further grows if we consider that the US allows for direct visualization of the nerve itself, enabling the assessment of any inter-individual anatomical variations. In this sense, Siegenthaler et al. evaluated the visibility of the target nerves and the variability of their course in relation to the fluoroscopically used bony landmarks, demonstrating how the medial branches and their relation to bony targets were mostly visualized with US [43].

Furthermore, as the above-analyzed studies demonstrate, US-guided injections to treat CFJS guarantee a shorter duration of the procedure and a less invasive path for the needle to pass through the soft tissues: this translates into less discomfort for patients. In 2018, Ye et al. compared US-guided facet joint intra-articular injections with CT-guided injections in 40 patients suffering from mid-to-low cervical spine pain. They found that US guidance had a higher first-attempt accuracy (100% versus 35%) and a shorter procedure time (6 min versus 14 min). Similarly, a retrospective observational study showed that US-guided MBB required less time (221 s versus 383 s) and fewer needle passes (two versus five) compared to FL-guidance [44]. Finally, US guidance allows for a change in trajectory and to adapt to any needs during the procedure.

Another important concern regards the greater availability of US compared to FL and CT devices; therefore, there is a greater possibility of accessing ultrasound-guided procedures for both patients and physicians. The outpatient settings for carrying out US-guided procedures are simple and can easily be transferred, unlike what happens for FL- and CT-guided techniques.

On the other hand, US-guided procedures are cheap, simple to learn and to carry out. However, a disadvantage compared to the injections guided by ionizing radiation is that the US-guided infiltration is more operator-dependent, therefore, requiring greater skill and experience, and a long learning curve [45,46]. An expert committee determined that the level of difficulty of cervical medial branch block procedures was “level III (advanced)”. Therefore, a great deal of practice is required to carry out ultrasound-guided cervical medial branch block [47]. Kwon et al. used a cervical spine phantom to help beginners improve their proficiency in performing US-guided CMBB. The most common mistake made by beginners during US-guided injections was failing to visualize the needle during insertion [48]. This is a clear demonstration that US practice must be carried out consistently and under the supervision of expert operators.

Finally, sometimes the patient’s body habitus, such as obesity, may limit the effectiveness of US. In such cases, FL and US can be used complementarily.

Even if it was not among the aims of this study, from the reviewed articles a good safety profile also emerges for all the US-guided procedures used for the treatment of CFJS. No significant side effects or reactions to US-guided procedures were reported. Relative contraindications to interventional techniques were described in patients receiving treatment with antithrombotic agents and anticoagulants. Prior to cervical facet joint interventions, patients on warfarin therapy must have their prothrombin time (PT) checked and documented to be at acceptable levels. The combination of multiple drugs with aspirin and nonsteroidal anti-inflammatory drugs or other antiplatelet therapy may be considered to increase the risk of spinal hematoma.

A key issue that needs to be addressed is how to design and conduct future studies in this field. There is a pressing need for further research involving larger sample sizes to provide robust, evidence-based knowledge that benefits both the scientific community and practicing physicians who require reliable data to guide patient care. It is also essential to ensure homogeneity in the study population to avoid potential confounding results. Calculating the appropriate sample size is crucial for detecting clinically significant differences between groups.

Carefully defining eligibility criteria, as well as properly managing randomization and allocation of participants, is vital. For instance, random sequence generation and allocation concealment can be effectively managed by using sealed envelopes provided by a blinded investigator or by employing computerized random generator software (e.g., randomizer.at or randomizer.org, accessed on 1 August 2024). Performance bias can be reduced by blinding investigators and using standardized equipment across all groups, such as covered 5 mL syringes, and masking the solutions injected into the CFJs.

It is also important to report all measured outcomes, emphasizing statistically significant results and including effect size calculations to facilitate a clear understanding of the findings. To minimize potential confounding factors, it is advisable to assess pain and NSAID use before the procedure, ensure that patients are thoroughly instructed on questionnaire completion, and provide clear imaging captured by the sonographer to verify procedural success.

By following this framework, future studies can achieve better reproducibility and generalizability of results, while minimizing the various biases that encountered in previous research.

This study is not free from limitations. Despite efforts to screen high-quality studies, only two of the nine studies reviewed were found to have a low risk of bias according to quality assessment. Six studies had a moderate risk of bias, and one had a high risk of bias. This was largely due to the limited literature available on this specific subject. Additionally, the studies analyzed were highly heterogeneous in terms of intervention, methodology, and outcomes, making it challenging to compare the results. Furthermore, regarding the selection of trials, by choosing to include only studies published in the last ten years, there is a potential risk of excluding earlier high-quality research that could have contributed valuable insights. Nevertheless, this exclusion criterion was deemed necessary to ensure that the review reflects the most recent advancements and current trends, with the aim to provide an up-to-date perspective on the state of the art on this topic, which is crucial for informing current practice and future research directions.

## 5. Conclusions

CFJS is a common cause of chronic neck pain and motor disability. US-guided injections are a safe and effective method to treat this musculoskeletal disease, granting a high functional recovery and long-lasting pain relief, net of the used drugs. These procedures have the same accuracy and effectiveness as the FL- and CT-guided ones but they involve lower costs, shorter times and less tissue invasiveness. However, US-guided injections are strictly operator-dependent and require important training and solid practice to acquire good expertise. Clinics and healthcare institutions could invest in comprehensive training programs to enhance clinicians’ proficiency in performing US-guided injections, considering the economic advantages of this method on medical expenses and its effectiveness. The impact could also directly benefit patients, as using this less invasive method may reduce intervention time and patient stress, while also increasing accessibility to the treatment, which can be performed in an outpatient setting.

Future studies should further explore the potentials of this procedure in terms of effectiveness and safety, so as to increase the supporting scientific evidence and allow it to have a more prominent place in the international guidelines for CFJS management.

## Figures and Tables

**Table 1:** Summary characteristics of the selected studies. Cervical facet joints (CFJ); Ultrasound (US); fluoroscopy (FL); computed tomography (CT); cervical medial branch blocks (CMBBs); Cervical Facet Joint Syndrome (CFJS); Body mass index (BMI); Numerical Rating Scale (NRS); interquartile range (IQR); ponderal index (PI); third occipital nerve block (TONB); Cervical range of motion (CROM); whiplash associated disorders (WAD); Neck Disability Index (NDI); Visual Numeric Scale (VNS); intra-articular blocks (IAB); Medial branch blocks (MBB); nociceptive flexion reflex (NFR); cranio-cervical flexion test (CCFT); Leeds Assessment of Neuropathic Symptoms and Signs (s-LANSS); Posttraumatic Diagnostic Scale (PDS); Pain Catastrophizing Scale (PCS); International Unit (IU).

Authors	Sample	Research Design	Collected Data and Outcomes	Results
Bodor et al. (2022) [24]	60 facet joints in 36 patients with chronic cervical facet joint syndrome (CFJS).	Type of Study: Cohort study. Objective: To determine the accuracy of ultrasound-guided cervical facet joint injections using a lateral technique and to describe the technique. Contrast dye was used to confirm it be intra-articular. Drugs: A mixture of 0.3 to 0.5 mL ropivacaine 0.5% plus 5 to 10 mg of triamcinolone acetonide 40 mg/mL was injected. Anatomical targets: facet joints C2-C3; C3-C4; C4-C5; C5-C6.	Data collected included: type of contrast pattern, Age, Sex, facet joint level, body mass index (BMI) and ponderal index (PI).	The accuracy of ultrasound-guided cervical facet joint injections using the lateral technique ranged from 92% to 98%.
Cohen et al. (2010) [25]	24 patients with axial (neck arm) cervical pain received cervical medial branch block (CMBB).	Type of Study: Randomized double-blinded trial, 50% of the patients in each group were sub-allocated to receive the blocks in the prone position and the other 50% through a lateral approach. Objective: The objective of this study was to evaluate the accuracy of medial branch blocks by using different injectate volumes. Participants then underwent computed tomography (CT) of the cervical spine to evaluate accuracy. Drugs: A mixture of either 0.5 or 0.25 mL of bupivacaine mixed with contrast was used. Anatomical targets: cervical medial branch.	Data collected: Age, sex, duration of pain, block level, obesity, n° patients on opioid therapy. Outcomes: baseline median Numerical Rating Scale (NRS) score-interquartile range (IQR), baseline median NDRI score (IQR), postblock median NRS score (IQR).	16 instances of aberrant spread were observed in 9 patients receiving blocks using 0.5 mL versus 7 occurrences in 6 patients in the 0.25 mL group. Foraminal spread occurred in 5 instances using 0.5 mL and in 2 cases with 0.25 mL. No significant difference in any outcome measure was observed between the prone and lateral positions. The two techniques were found to be equally effective in reducing pain.
Finlayson et al. (2013) [26]	40 patients with CFJS	Type of Study: Randomized observer-blinded trial. Patients undergoing third occipital nerve block (TONB) were randomized to FL or US guidance. Objective: Comparison between 2 different guidance modalities (FL, US). Drugs: A mixture of local anesthetic and radiographic contrast was injected in both groups. Anatomical targets: Third Occipital nerve (TON).	Data collected: Age, sex, Body Mass Index (BMI) Outcomes: pre-block NRS score, performance time, success rate, pain levels before and after block, area of sensory hypoesthesia, quality of the block (assessed by electrical perceptual threshold), procedure-related complications.	FL and US guidance provide similar success rates for TONB. US is associated with greater efficiency (decreased performance time, fewer needle passes).
Finlayson et al. (2015) [27]	50 patients with CFJS referral patterns elicited from C7 medial branch stimulation	Type of Study: Randomized observer-blinded trial. Patients undergoing C7 CMBB randomized to FL or US guidance. Objective: US, using a biplanar imaging technique, could provide a shorter performance time than conventional FL for C7 cervical medial branch blocks. Drugs: A 0.6-mL mixture of local anesthetic and radiographic contrast was injected in both groups. Anatomical targets: C7 CMBB.	Data collected: Age, sex, BMI. Outcomes: pre-block NRS score, performance time, success rate, pain levels before and after block, incidence of aberrant spread and procedure-related complications.	US-guided using a biplanar approach provides a similar success rate to FL for C7 cervical medial branch blocks. US is associated with a higher efficiency (decreased performance time and fewer needle passes) in the comparison with FL-guided technique.
Obernauer et al. (2013) [28]	40 adult patients with chronic neck pain.	Type of Study: Prospective randomized trial. Patients were assigned to one of two groups by chance; one group underwent US-guided infiltrations (US group), the other group underwent CT guided injections (CT group). Objective: To evaluate accuracy, time-saving, radiation doses and pain relief of US-guided facet joint injections versus CT-controlled interventions in the cervical spine. Drugs: 1 mL mixture of betamethasone (4 mg/mL); and Bupivacaine hydrochloride (0.25%, 2.5 mg/mL). Anatomical targets: facet joint of the middle and lower cervical spine(C2-C3; C7-D1) for CMBB.	Data collected: Gender, Age, BMI. Outcomes: Visual Analogue Scale (VAS), accuracy, time to final needle placement, dose of radiation.	US-guided intra-articular injections show the same therapeutic effect as CT-guided intra-articular injections and result in a significant reduction in procedure duration without any radiation exposure.
Park et al. (2016) [29]	186 patients with chronic CFJS.	Type of Study: retrospective comparative study, 68 patients received US-guided and 58 patients received FL-guided CMBB. Objective: To compare the mid-term effects and advantages of the US-guided versus the FL-guided CMBBs for chronic CJFS assessing pain relief, functional improvement, and injection efficiency. Drugs: 1 mL of a mixture of 1% lidocaine (0.5 mL) and dexamethasone (5 mg/mL; 0.5 mL). Anatomical targets: facet joints of the middle and lower cervical spine (C2-C3; C7-D1) for CMBB.	Data collected: Sex, Age, BMI, injection method, number of injections, analgesic use, pain duration. Outcomes: Neck Disability Index (NDI), Visual Numeric Scale (VNS) score.	Both NDI and VNS scores showed improvements at 1, 3, and 6 months after the last injection in both groups, with no significant differences between the groups. US-guided procedure was associated with shorter administration duration and fewer needle passes.
Park et al. (2012) [13]	400 patients with long-standing cervical myofascial pain referred to CFJS over a period of 6 months.	Type of Study: Randomized controlled clinical trial. Patients were divided into two groups of 200 each: group 1 and group N (non-injection). Group 1 also received therapeutic CFJ injections at bilateral C5/C6 and C6/C7 levels after double-blind controlled diagnostic blocks. Objective: To investigate the effects of therapeutic CFJ injections on patients with myofascial pain referred to CFJS. Drugs: local anesthetic blocks using 0.3 mL of 1% lidocaine and 0.25% bupivacaine; then injection using a mixture of 0.5 mL of 1% lidocaine, 5 mg of triamcinolone and 187.5 IU of hyaluronidase. Anatomical targets: CFJ bilateral C5/C6 and C6/C7.	Data collected: Comorbidities, presence of tension-type headache, Age, Sex, Treatment duration, symptom-free period. Outcomes: Cervical range of motion (CROM), NRS.	During the follow-up, group 1 exhibited a greater CROM, a greater mean NRS pain reduction, and a lower incidence of combined tension-type headache than group N. The treatment cycle for younger patients in group 1 was shorter and they experienced a longer period without symptoms.
Pasuhirunnikornt al. (2023) [30]	62 patients with chronic CFJS.	Type of study: Prospective, double-blinded study. Objective: To compare the clinical effect of the CMBB using lidocaine versus bupivacaine. Drugs: For CMBB, either 2% lidocaine or 0.5% bupivacaine with a volume of 0.5–1 mL per level. Anatomical targets: Cervical medial branches.	Data collected: Age, gender, referred pain area, BMI, side (right/left), procedure levels, complications. Outcomes: pain intensity (NRS), NDI, duration of pain reduction by at least 50% (in months).	Clinical benefits were achieved using both lidocaine and bupivacaine in the CMBB. No significant difference in the duration of 50% pain relief, but lidocaine yielded better performance in pain reduction in both early and later follow-up after the intervention. No differences in terms of safety and accuracy between the two drugs were reported. Lidocaine could be considered the best local anesthetic due to its better performance.
Smith et al. (2013) [31]	90 patients with chronic whiplash-associated disorders (WAD)	Type of study: A cross-sectional comparison study with 3 groups: 58 who responded to cervical facet block procedures (WAD_R); 32 who did not respond (WAD_NR); 30 Healthy Controls (HC)s. Objective: to compare the clinical manifestations after treatment with facet blocks (FB), intra-articular blocks (IAB) or comparative medial branch blocks (MBB), in two WAD groups and the healthy control group. Drugs: IAB: injection of 0.5 cc of local anaesthetic (1% Bupivicaine) and 0.5 cc of corticosteroid. MBB: 0.5 cc of 2% Lidocaine. Anatomical targets: Cervical facet joint, Cervical medial branch.	Data collected and Outcomes: quantitative sensory tests (pressure; thermal pain thresholds; brachial plexus provocation test); nociceptive flexion reflex (NFR); motor function (CROM); activity of the superficial neck flexors during the cranio-cervical flexion test (CCFT). Self-reported measures were gained from the following questionnaires: neuropathic pain (s-LANSS); psychological distress (General Health Questionnaire-28), post-traumatic stress (PDS) and pain catastrophization (PCS).	Both chronic WAD responders and non-responders to facet block procedures exhibit a shared pattern of sensory disturbance, motor dysfunction, and psychological distress. Higher levels of pain catastrophizing and increased medication intake were found in the WAD_NR group.

**Table 2:** The modified version of Jadad quality scores for the selected studies.

Study	Was the Treatment Randomly Allocated?	Was the Randomization Procedure Described and Appropriate?	Was There a Description of Withdrawals and Dropouts?	Was There a Clear Description of the Inclusion/Exclusion Criteria?	Were the Methods of Statistical Analysis Described?	Jadad Score (0–5)
Bodor et al. (2022) [24]	No	No	Yes	Yes	No	2
Cohen et al. (2010) [25]	Yes	Yes	Yes	Yes	Yes	5
Finlayson et al. (2013) [26]	Yes	Yes	Yes	No	Yes	4
Finlayson et al. (2015) [27]	Yes	Yes	No	No	Yes	3
Obernauer et al. (2013) [28]	No	No	Yes	Yes	Yes	3
Park et al. (2012) [13]	No	Yes	Yes	No	Yes	3
Park et al. (2016) [29]	No	No	Yes	Yes	Yes	3
Pasuhirunnikorn et al. (2023) [30]	Yes	Yes	Yes	Yes	Yes	5
Smith et al. (2013) [31]	No	No	Yes	Yes	Yes	3

## Data Availability

The original contributions presented in the study are included in the article and further inquiries can be directed to the corresponding author.
